# Pirfenidone use in fibrotic diseases: What do we know so far?

**DOI:** 10.1002/iid3.1335

**Published:** 2024-07-05

**Authors:** Aldo Torre, Froylan David Martínez‐Sánchez, Sofía Mercedes Narvaez‐Chávez, Mariana Ariel Herrera‐Islas, Carlos Alberto Aguilar‐Salinas, Jacqueline Córdova‐Gallardo

**Affiliations:** ^1^ Metabolic Unit Instituto Nacional de Ciencias Médicas y Nutrición “Salvador Zubiran” Mexico City Mexico; ^2^ Facultad de Medicina Universidad Nacional Autonoma de Mexico Mexico City Mexico; ^3^ Department of Internal Medicine Hospital General “Dr. Manuel Gea González” Mexico City Mexico; ^4^ Department of Hepatology Hospital General “Dr. Manuel Gea González” Mexico City Mexico

**Keywords:** chronic liver disease, fibrosis, inflammation, Pirfenidone

## Abstract

**Background:**

Pirfenidone has demonstrated significant anti‐inflammatory and antifibrotic effects in both animal models and some clinical trials. Its potential for antifibrotic activity positions it as a promising candidate for the treatment of various fibrotic diseases. Pirfenidone exerts several pleiotropic and anti‐inflammatory effects through different molecular pathways, attenuating multiple inflammatory processes, including the secretion of pro‐inflammatory cytokines, apoptosis, and fibroblast activation.

**Objective:**

To present the current evidence of pirfenidone's effects on several fibrotic diseases, with a focus on its potential as a therapeutic option for managing chronic fibrotic conditions.

**Findings:**

Pirfenidone has been extensively studied for idiopathic pulmonary fibrosis, showing a favorable impact and forming part of the current treatment regimen for this disease. Additionally, pirfenidone appears to have beneficial effects on similar fibrotic diseases such as interstitial lung disease, myocardial fibrosis, glomerulopathies, aberrant skin scarring, chronic liver disease, and other fibrotic disorders.

**Conclusion:**

Given the increasing incidence of chronic fibrotic conditions, pirfenidone emerges as a potential therapeutic option for these patients. However, further clinical trials are necessary to confirm its therapeutic efficacy in various fibrotic diseases. This review aims to highlight the current evidence of pirfenidone's effects in multiple fibrotic conditions.

## Graphical abstract

Pirfenidone has several pleiotropic and anti‐inflammatory mechanism of action through different molecular pathways. Consequently, it attenuates multiple inflammatory processes, including the secretion of proinflammatory cytokines, apoptosis, and fibroblast activation.

## 1 INTRODUCTION

Pirfenidone is an antifibrogenic and anti‐inflammatory drug extensively tested for idiopathic pulmonary fibrosis and currently under investigation for various other fibrotic diseases.^1^ Both animal and human models have demonstrated that pirfenidone exerts multiple beneficial effects through its anti‐inflammatory properties, including reducing proinflammatory cytokine secretion, decreasing the recruitment of proinflammatory cells, and inhibiting fibroblast activation, which consequently leads to reduced collagen deposition.^1^, ^2^ Several clinical trials are currently underway to confirm these effects in various organs.

## 2 PHARMACOKINETICS AND METABOLIC ACTIVATION OF PIRFENIDONE

Pirfenidone has been shown to exert anti‐inflammatory and antifibrotic effects through different pathways.^2^, ^3^ Pirfenidone is primarily metabolized by cytochrome P450 (CYP1A2), producing 5‐hydroxymethyl pirfenidone and 5‐carboxy pirfenidone. The methyl group is oxidized to form 5‐hydroxymethyl pirfenidone, which is further oxidized to the more polar 5‐carboxy pirfenidone. Additionally, both pirfenidone and 5‐carboxy pirfenidone undergo glucuronidation, forming various metabolites. P450 enzymes play a crucial role in oxidative metabolism and metabolic activation of pirfenidone, with CYP3A4 being the major contributor, followed by CYP2A6 and CYP1A2. The expression of CYP1A2 is particularly important for sulfation‐mediated metabolic activation.^2^ The phase II conjugation pathway involves catabolization by sulfotransferases (SULTs). SULTs catalyze the transfer of a sulfonyl group from 3’‐phosphoadenosine‐5’‐phosphosulfate to an amino, hydroxyl, or sulfhydryl group of substrates. This process tends to enhance hydrophilicity, thereby increasing the bioavailability of pirfenidone.^2^

The recommended oral dose for immediate release pirfenidone is 801 mg per day during the first week, increasing to 1602 mg per day in the second week, and finally to 2403 mg per day starting from the 15th day onwards. For extended‐release pirfenidone, the recommended dose is 600 mg per day during the first week, increasing to 1200 mg per day in the second week, and reaching a final dose of 1800 mg per day in the third week.^4^, ^5^

In a matrix‐assisted desorption/ionization‐mass spectrometry imaging study conducted on female mice, pirfenidone (5‐methyl‐N‐phenyl‐2‐1H‐pyridone‐d5) was dissolved in carboxymethylcellulose and administered orally at a dose of 500 mg/kg.^6^ Pirfenidone was found to be highly abundant in the lung, kidney, and liver. Both 5‐hydroxymethyl pirfenidone and 5‐carboxy pirfenidone showed heterogeneous distribution patterns in the lung and kidney, with 5‐hydroxymethyl pirfenidone primarily localized along the main bronchi and predominantly in the medulla of the kidney. The liver reached its maximum concentration at 30 min, followed by the kidney at 45 min. The liver also showed the highest maximum concentration and the highest area under the curve due to the first‐pass effect. For immediate release pirfenidone, the time to maximum concentration was 30 min, with a half‐life of 46 min in the liver, 67 min in the lungs, and 49 min in the kidneys.^6^ These findings demonstrate the rapid absorption, metabolism, and excretion of pirfenidone.

Studies in mice and humans conducted in vitro have demonstrated that the pirfenidone metabolites 5‐hydroxymethyl and 5‐carboxy pirfenidone are most abundant in fibrotic lungs.^7^ However, in fibrotic lungs, these metabolites were predominantly localized in less affected areas, suggesting that the distribution and possibly the metabolism of pirfenidone are increased in fibrosis. Accumulation in affected areas may be necessary for its antifibrotic effects.^7^ These metabolites inhibit transforming growth factor (TGF)‐β‐induced collagen synthesis, thereby demonstrating their antifibrotic activities.^7^, ^8^

## 3 MOLECULAR MECHANISMS OF ACTION OF PIRFENIDONE

**Figure 1:**
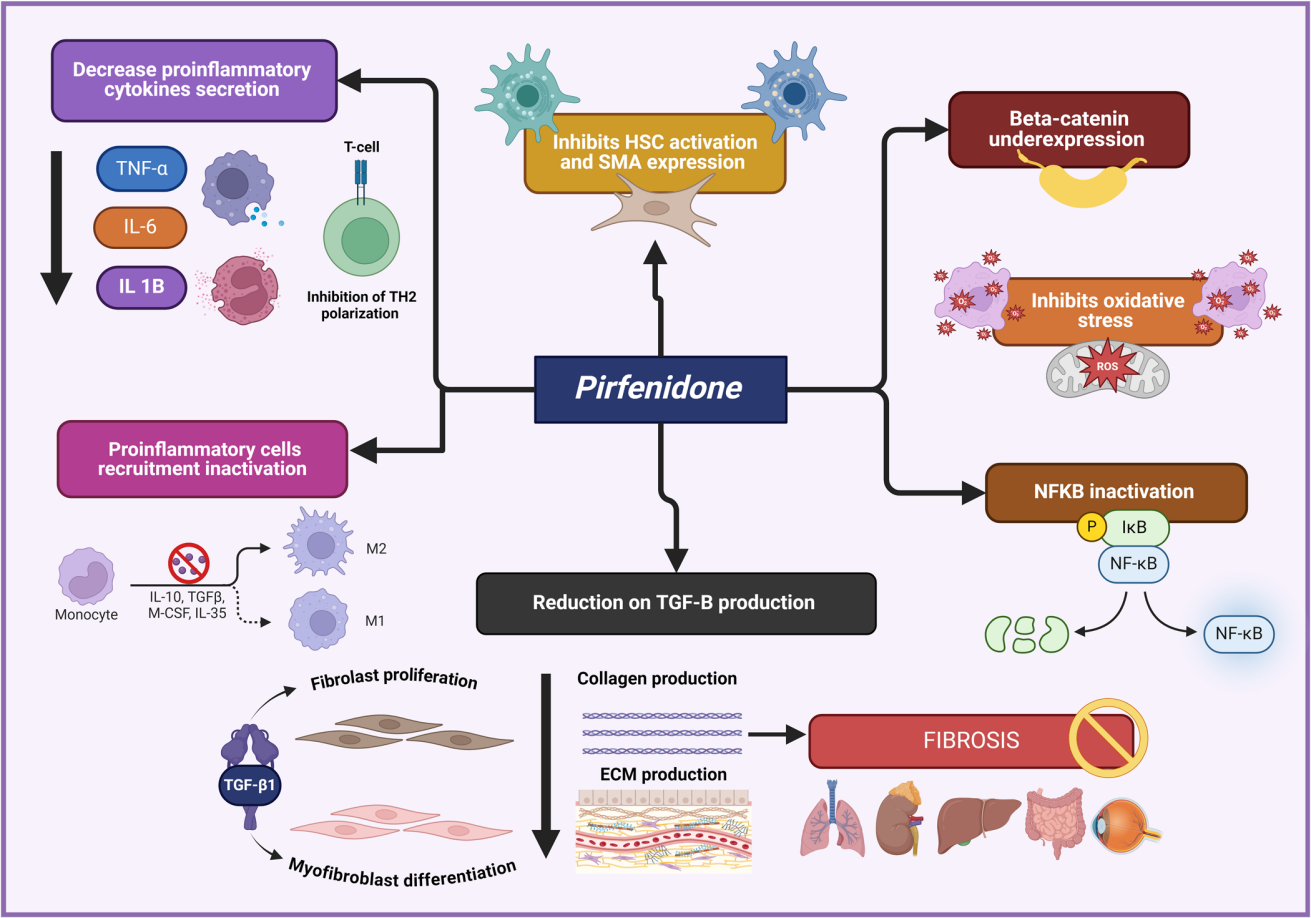
Molecular mechanisms of action of pirfenidone. PFD downregulates the expression of TGF‐β by reducing its profibrotic activity. It inhibits TGF‐β signaling and decreases the transcription of genes for collagen synthesis and extracellular matrix (ECM) production. It also reduces the production of proinflammatory cytokines such as tumor necrosis factor‐alpha (TNF‐α), interleukin‐1 beta (IL‐1β), and interleukin‐6 (IL‐6), declining fibrosis formation. PFD reduces oxidative stress by scavenging free radicals and diminishing levels of reactive oxygen species (ROS). It affects signaling pathways such as the Smad pathway, which is downstream of TGF‐β, leading to a decrease of fibrogenic activity. Consequently, it inhibits fibroblast proliferation and increases fibroblast apoptosis.

Pirfenidone has been suggested as a safe option to slow or inhibit the progression of fibrotic lesions and prevent the formation of new ones after tissue injury (Figure 1).^3^ Pirfenidone's anti‐fibrotic role is well‐demonstrated, as it inhibits the overexpression of type I collagen induced by TGFβ1 and heat shock protein 47 (HSP47) in A549 cells.^8^ Notably, pirfenidone exhibits anti‐inflammatory properties by primarily reducing the secretion of proinflammatory cytokines such as TNF‐alpha, IL1B, and IL6 by circulating macrophages, neutrophils, and endothelial cells.^3^, ^5^ These cytokines induce various local and systemic responses, resulting in increased recruitment of inflammatory cells from the bloodstream to the site of inflammation.^8^, ^9^

Injuries often trigger an intense inflammatory response, resulting in significant tissue damage and necessitating anti‐inflammatory intervention. Various blood cells, including macrophages, play crucial roles in this process, initiating and regulating both innate and adaptive responses. Activated macrophages can assume different phenotypes, with M1 promoting tissue damage and M2 aiding in tissue repair. Pirfenidone has been shown to modulate the activity of both M1 and M2 macrophages, reducing the expression of proinflammatory cytokines and markers associated with inflammation. Notably, pirfenidone's protective effects extend to the kidney, where it inhibits cell death by preventing the activation of caspase‐9 and caspase‐3.^10^, ^11^ Activated macrophages can be categorized into M1 (inhibiting cell proliferation and causing tissue damage with a Th1‐like phenotype) and M2 (promoting cell proliferation and tissue repair with a Th2‐like phenotype).^10^ Studies in murine models have highlighted pirfenidone's ability to significantly reduce the expression of M1 macrophages, known for their secretion of proinflammatory cytokines like TNF‐alpha and IL6, as well as nitric oxide synthase (NOS) 2. ^10^, ^11^ Additionally, pirfenidone leads to a decrease in the levels of arginase‐1, dectin‐1, CD206, and CD86 expressed on M2 macrophages. Notably, in the kidney, pirfenidone prevents the translocation of cytochrome C from mitochondria to the cytosol, thereby inhibiting key players in the process of cell death, including caspase‐9 and caspase‐3.^12^

Pirfenidone's anti‐inflammatory effects are evident through its reduction of TNF‐alpha and IL‐6 levels in lung tissues, inhibiting epithelial‐mesenchymal transition and lung fibrosis in murine silicosis models.^11^ These effects may be mediated through the TGFβ‐1/Smad pathway, where TGF‐β1 binds to its receptor, TGFβ‐R, forming a transmembrane complex to exert fibrogenic actions.^10^, ^11^ Additionally, TGF‐β suppresses fibroblast‐mediated fibrotic processes by reversing the regulation of lung fibroblast activity. Treatment with pirfenidone significantly decreased Col‐III, alpha‐ smooth muscle actin (SMA), and fibronectin protein levels in lung tissues, demonstrating its efficacy in alleviating inflammation and reducing extracellular matrix deposition. Pirfenidone also directly downregulated the release of TNF‐alpha and IL‐6, further contributing to its anti‐inflammatory effects. Moreover, pirfenidone acts on the WNT pathway by phosphorylating and degrading Beta‐catenin through GSK‐3 beta.^10^

Pirfenidone's effects extend beyond macrophages to T cells, where it inhibits the proliferation response of T‐cell receptor (TCR)‐stimulated CD4+ cells both in vitro and in vivo.^12^ This compound also reduces the numbers of both CD4 and CD8 cells. In the liver, Type 2 T helper (Th2) cells have been implicated in liver fibrosis. Navarro‐Partida et al. demonstrated in Wistar rats that pirfenidone induces the downregulation of Th2 transcription factors and proteins such as GATA3 and IL4.^13^ This suggests that pirfenidone may modulate and restrict the profibrogenic Th2 response by inhibiting p38 and upregulating GATA3.^13^ Additionally, pirfenidone impacts B lymphocytes by inhibiting the secretion of inflammatory cytokines and neutrophil chemoattractants induced by cytokines and iNOS, achieved through the inhibition of NF‐κB activation.^14^

Pirfenidone's impact extends to fibroblasts, as evidenced by studies on various cell types. Cui et al. demonstrated its effects on colonic fibroblasts in inflammatory bowel disease, showing that pirfenidone suppresses motility, reduces fibroblast metabolic activity, and diminishes collagen production.^15^ Similarly, in cardiac fibroblasts, pirfenidone inhibits the expression of alpha‐SMA, thereby impeding the transformation of fibroblasts into myofibroblasts and contributing to fibrosis.^16^ Topical pirfenidone in a murine model has been shown to inhibit proliferation, migration, and collagen synthesis of Tenon capsule fibroblasts (human orbital fibroblasts).^17^ Local pirfenidone application prevents fibrogenesis by inhibiting Smad 2/3 nuclear translocation and regulating TGF‐β1 protein phosphorylation, leading to the downregulation of mesenchymal markers in human retinal pigment epithelial cell line ARPE‐19.^1^ Additionally, it inhibits cell proliferation, migration, and epithelial‐mesenchymal transition in the human lens epithelial cell line SRA01/04.^1^, ^17^ In patients undergoing lung transplantation, pirfenidone interferes with fibroblast migration and activation through the Hedgehog pathway.^18^ Specifically, it blocks fibroblast migration, proliferation, and the transcription factor GLI2 within the Hedgehog pathway, which is reactivated in the adult lung after injury.^18^, ^19^ In pulmonary fibrosis, GLI1 is upregulated in fibroblasts and myofibroblasts. GLI proteins potentially contribute to pulmonary fibrosis development through various mechanisms, including controlling the expression of proliferative and antiapoptotic genes, regulating collagen production, inducing Snail expression to promote epithelial‐to‐mesenchymal transition, and modulating the transcriptional activity of the alpha‐SMA gene to promote fibroblast‐to‐myofibroblast differentiation.^18^, ^19^ It has been demonstrated that pirfenidone interferes with GLI2 transcription factor activity, affecting not only the Hedgehog pathway but also TGFβ and other signaling systems.^19^ Research confirms pirfenidone inhibits the production and release of pro‐fibrotic and proinflammatory cytokines like TGF‐β, tumor necrosis factor‐alpha, and interleukin IL‐6, thereby delaying fibroblast proliferation and collagen deposition.^10^ Nevertheless, the precise mechanism by which pirfenidone inhibits fibrosis is not fully understood.

According to previous studies, pirfenidone inhibits the growth factor‐dependent regulation of airway fibroblast proliferation and downregulates arginase, an essential enzyme for collagen synthesis.^8^, ^20^ Treatment with pirfenidone resulted in the inhibition of arginase, possibly due to the inhibition of TGFβ, which is a potent inducer of arginase‐inducing cytokines. The indirect effect of downregulating arginase may also involve decreased nitric oxide production.^20^, ^21^

Pirfenidone exhibits an inhibitory effect on angiogenesis by suppressing the protein levels of VEGF‐A, VEGFR‐2, and NRP‐1, notably at a concentration of 0.6 g/ml.^21^ When compared to ranibizumab, a monoclonal antibody known for inhibiting VEGF‐A and VEGFR‐2 expression, both drugs down‐regulate the protein expression of VEGF‐A, VEGFR‐2, and NRP‐1 in human umbilical vein endothelial cells (HUVECs), with no significant statistical difference observed. Pirfenidone also attenuates HUVEC proliferation, viability, migration, invasion, and tube formation, displaying low cytotoxicity. Its mechanism of action may involve the VEGF‐A/VEGFR‐2 pathway and its downstream AKT signaling, contributing to antiangiogenic effects.^8^, ^10^, ^21^ These findings suggest that pirfenidone may inhibit the wound healing process through anti‐inflammatory, anti‐fibrotic, and antiangiogenic mechanisms. Other studies support pirfenidone's role as a multi‐targeted anti‐scarring agent, indicating its potential in regulating wound healing.^21^

## 4 POTENTIAL ADVERSE EFFECTS

Pirfenidone's safety profile has been extensively evaluated in clinical trials. Table 1 shows the main adverse events of pirfenidone, incidence and recommendations for management. Reported adverse effects include gastrointestinal issues such as nausea (36%), diarrhea (29%), dyspepsia (19%), and vomiting (14%).^22^, ^23^, ^24^, ^25^, ^26^, ^27^ Most were mild to moderate, transient, and improved with prokinetic agents. Skin‐related effects like rash (32%) and photosensitivity (12%) have also been documented. Mild to moderate aminotransferase elevations, rarely associated with bilirubin increase, were observed, prompting recommendations for liver function tests before and during treatment.^27^ Adverse effects led to discontinuation in 1%–3% of cases, mainly due to skin rashes or severe nausea.^25^ Treatment should be administered in experienced centers. The recommended adult dose is 267 mg three times daily with meals, gradually increasing to a maximum of nine capsules per day to manage side effects.^22^, ^23^ Barranco‐Garduño et al.^4^ compared the pharmacokinetics of immediate‐release and extended‐release formulations, finding similar exposure profiles but fewer concentration fluctuations and longer mean residence times with pirfenidone extended‐release. This suggests that prolonged‐release formulations could reduce adverse events and improve adherence. The PROMETEO Study in Advanced Liver Fibrosis also found that extended‐release pirfenidone had few side effects and no significant liver function test alterations.^28^ Table 1 summarizes the main adverse events related to pirfenidone.^29^, ^30^, ^31^, ^32^, ^33^, ^34^

**Table 1:** Main adverse events of pirfenidone, incidence and recommendations for management.

Adverse Events (AEs)		Reported incidence	Recommendations for management	Dose reduction or drug holiday recommended	In case of persistent/severe AEs: Discontinue and consider treatment with another anti‐fibrotic.
Gastrointestinal	Abdominal pain	24%	Take the dose with food/meal Proton Pump Inhibitor (PPI) can be prescribed	yes	Yes
	Diarrhea	26%	Take the dose with food/meal Loperamide and atropine/diphenoxylate can be prescribed	Yes	Yes
	Dyspepsia	19%	PPIs and antacids like aluminum and magnesium hydroxide and calcium carbonate may be prescribed	Yes	Yes
	Vomiting	13%	PPIs and 5HT3 receptor antagonists are recommended	Yes	Yes
	Gastroesophageal Reflux Disease	11%	PPIs and H2 (histamine 2) blockers	Yes	Yes
Skin/Dermatological	Rash	30%	Ensure the use of sun protection (e.g., protective clothing, sunscreen, long sleeves, and hats) as well as reduce the amount of sun exposure.	Yes	Yes
Musculoskeletal	Arthralgia	10%	Assess for another causes. Vitamin D and bone density testing.	No	No
Other	Upper respiratory tract infections (URTI)	27%	URTI should be monitored and treated with supportive care, over the counter medications and/or antibiotics.	No	No
	Dizziness	18%	Evaluate for other causes. Blood tests should be ordered, and levels of magnesium should be checked.	No	No
	Anorexia	13%	Add oral supplementation.	Yes	Yes
	Insomnia	10%	Assess for another causes of insomnia. Sleep hygiene	No	No
	Weight loss	10%	Increase the frequency and size of meals, encourage eating and discuss eating habits Fatty meals.	Yes	Yes
	Fatigue	26%	Other causes of fatigue should be ruled out.	Yes	Yes

## 5 EVIDENCE OF PIRFENIDONE USE IN FIBROTIC DISEASES

### 5.1 Idiopathic pulmonary fibrosis and other pulmonary fibrotic diseases treatment

Idiopathic pulmonary fibrosis (IPF) is a progressive and aggressive lung disease of unknown etiology, characterized by a gradual decline in lung function.^35^ The efficacy of pirfenidone in improving prognosis and reducing exacerbations and mortality in IPF has been extensively studied.^35^ A recent meta‐analysis found that pirfenidone, compared to placebo, did not significantly improve acute exacerbations of IPF (RR 0.59, CI 0.19–1.84), but it did improve worsening IPF (RR 0.84, CI 0.74–0.85). Pirfenidone also reduced the risk of a > 10% decline in forced vital capacity (FVC) (RR 0.63, CI 0.41–0.85) and improved 6‐min walk test distance (RR 0.74, CI 0.64–0.86). These benefits were not associated with serious adverse effects, only mild ones such as photosensitivity and changes in aminotransferases.^35^ The CAPACITY trial confirmed that pirfenidone has a favorable benefit‐risk profile, making it a viable therapeutic option for IPF patients.^36^ Additionally, pirfenidone use was associated with reduced all‐cause mortality (HR 0.28, 95% CI 0.23–0.86) and fewer instances of ≥10% FVC decline or airway‐related hospitalization (HR 0.46, 95% CI 0.28–0.76), with an overall survival benefit and improved lung function.^37^, ^38^ Another meta‐analysis corroborated these findings, highlighting the drug's role in prolonging progression‐free survival and preserving lung function in IPF patients.^39^

Several trials have shown that treating IPF patients with pirfenidone for 52 and 120 weeks reduces both IPF‐related and all‐cause mortality, demonstrating a decrease in relative risk of mortality.^39^, ^40^ Markers associated with this mortality reduction include CCL3, CCL18, CXCL13, CXCL14, periostin, and YKL40, with CCL18 being a consistent predictor of disease progression and changes in FVC%.^41^ Based on these findings, pirfenidone is part of the treatment regimen for IPF patients with FVC between 50% and 80%. Guidelines recommend discontinuing treatment if there is a ≥ 10% decrease in FVC over 12 months, indicating disease progression.^42^ Preoperative use of pirfenidone in IPF patients undergoing surgeries under general anesthesia has been effective in reducing severe postoperative respiratory complications.^43^ In patients with IPF undergoing lung cancer surgical resection, pirfenidone has demonstrated safety as a prophylactic treatment and reduced acute postoperative exacerbations.^44^

Pirfenidone has been assessed in non‐IPF diseases with interstitial involvement and found to be well‐tolerated.^45^ Its effect was more pronounced in IPF patients with mild to moderate disease compared to other interstitial lung diseases (ILD).^45^ Another trial demonstrated that adding pirfenidone to standard therapy could attenuate disease progression.^46^ In rapidly progressive ILD related to amyopathic dermatomyositis, pirfenidone increased survival in patients with subacute ILD.^47^

In lung transplantation, pirfenidone reduced primary graft dysfunction, duration of mechanical ventilation, and the incidence of acute cellular rejection within the first 30 days.^48^ Further evidence from a study involving 11 patients indicated that pirfenidone was safe and slowed the rate of lung function deterioration in restrictive allograft syndrome.^29^ The latest update from the ATS/ERS/JRS/ALAT for managing idiopathic pulmonary fibrosis and progressive pulmonary fibrosis includes pirfenidone as part of the treatment for both conditions.^30^

### 5.2 Heart diseases

Pirfenidone has also demonstrated its anti‐fibrotic effect in the heart, making it another target organ.^31^ This mechanism is attributed to the inhibition of the activation of the TGFβ1/Smad3 signaling pathway.^32^ Pirfenidone achieves this antifibrotic effect by reducing platelet‐derived growth factor, matrix metalloproteinases, and proinflammatory mediators, improving mitochondrial function, modulating lymphocyte activation, and decreasing JAK2 and pSTAT3 expression in cardiac tissues; thereby attenuating cardiac hypertrophy.^33^, ^34^ In addition, pirfenidone contributes to a reduction in vascular permeability by inhibiting claudin 5 expression.^49^ Pirfenidone has demonstrated another significant result in left ventricular remodeling by reducing fibrosis through the inhibition of NLRP3 expression, attenuating the expression of IL‐1B in fibrotic and inflammatory pathways.^50^, ^51^ This action effectively prevents cardiac remodeling and collagen accumulation.^35^ In addition to these effects, pirfenidone also inhibits the AT1R/p38 MAPK pathways, correcting the RAS imbalance and increasing LXR‐alpha expression, resulting in a cardioprotective effect.^52^

Pirfenidone has been tested for coronary artery disease models.^53^ Nguyen et al. demonstrated that pirfenidone reduced total and non‐scarring fibrosis in rats with myocardial infarction, leading to reduced infarct scarring, improved left ventricular function, and decreased susceptibility to ventricular tachycardia.^54^ In murine cardiomyopathy models caused by increased afterload, pirfenidone reduced hypertrophy of ventricular myocytes, myocardial fibrosis, diastolic dysfunction, perivascular and interstitial fibrosis, and decreased the expression of TGF‐β, mineralocorticoid receptors, and natriuretic peptides.^33^ Pirfenidone had a broader action than standard drugs, significantly affecting pathways such as p38γ‐MAPK12 and TGFβ1‐SMAD2/3, and proteins like matrix metalloproteinase 2 and 14, PDGFA/B, and IGF1.^53^ A clinical trial by Lewis et al.^55^ confirmed pirfenidone's effect on cardiac fibrosis and inflammation. In a double‐blind phase 2 trial, they found that among heart failure patients with preserved ejection fraction and increased extracellular volume, pirfenidone treatment for 52 weeks reduced extracellular volume, indicating a reduction in myocardial fibrosis.

### 5.3 Glomerulosclerosis and other fibrotic kidney diseases

In the kidney, pirfenidone has demonstrated its efficacy in reducing tubulointerstitial fibrosis, like its effects in other organs, by inhibiting the activation of TGFβ1/Smad3 signaling pathways and downregulating miR‐21 expression.^56^, ^57^, ^58^, ^59^ Additionally, pirfenidone reduces mesangial matrix expansion and renal matrix gene expression by suppressing TGFβ activity, Smad2 and 3 phosphorylation.^60^ These pathways collectively down‐regulate renal fibroblast activation and proliferation. Pirfenidone's anti‐inflammatory effects have been effective in preventing chronic renal allograft dysfunction, reducing renal inflammation, and fibrosis.^61^ It also helps prevent ischemia by restoring nitric oxide production.^62^ Additionally, pirfenidone protects mitochondrial structures and functions by stabilizing the mitochondrial membrane and inhibiting the mitochondrial apoptotic signaling pathway. It increases superoxide dismutase levels, reducing oxidative stress and reactive oxygen species secretion.^63^

Acute kidney injury, a reversible reduction in renal function, was studied in a rat model to evaluate the effect of pirfenidone in improving renal function. Pirfenidone attenuates gentamicin‐induced acute kidney injury through the inhibition of the inflammasome‐dependent NLRP3 pathway in rats.^64^

Pirfenidone has shown promising effects in animal models of glomerulosclerosis, stabilizing renal function with significant improvements in inulin clearance and reducing renal cortical collagen accumulation.^59^ It suppressed collagen I, matrix metalloproteinase 2, and plasminogen activator inhibitor‐1 in the renal cortex, and exhibited an antiproteinuric effect, especially when combined with candesartan.^59^ In a remnant kidney rat model, Shimizu T et al. found that pirfenidone was significantly more effective than the control in stabilizing renal function at 12 weeks and reducing renal cortical collagen accumulation.^65^ In another rat model, pirfenidone attenuated interstitial fibrosis, decreased fibrotic markers and significantly reduced macrophage infiltration.^66^ Pirfenidone also demonstrated renoprotective effects, preventing elevations in plasma creatinine and blood urea nitrogen, reducing systolic blood pressure, and improving interstitial fibrosis in the renal cortex.^67^

An open‐label clinical trial with 21 patients with focal segmental glomerulosclerosis showed a 25% improvement in estimated glomerular filtration rate (eGFR) with pirfenidone treatment. The eGFR decline rate improved from a median of −0.61 ml/min/1.73 m^2^ at baseline to −0.45 ml/min/1.73 m^2^ during the 12‐month treatment period (*p* < .01), though pirfenidone had no effect on proteinuria.^68^ In patients with diabetic nephropathy, pirfenidone significantly preserved eGFR from baseline to the end of the study in the 1200 mg group compared to placebo.^69^

### 5.4 Liver fibrosis and other chronic liver diseases

Pirfenidone has demonstrated its anti‐fibrotic effects in the liver by reducing TGFβ1 levels and inducing regression of fibrosis in liver cirrhosis.^28^ This effect occurs through various pathways, including the reduction of concanavalin‐A‐induced hepatic inflammation by decreasing TNF‐alpha, TGF‐β, and TIMP‐1. Additionally, pirfenidone decreases collagen deposition and increases metalloproteinases such as MMP2.^70^ It also interacts with the renin‐angiotensin‐aldosterone system, impacting TGF‐β profibrotic pathways and activating several fibrotic mechanisms.^71^ In nonalcoholic steatohepatitis (NASH) models, pirfenidone's anti‐inflammatory effect, which inhibits TNF‐alpha, has been shown to decrease the activation of fibrotic pathways and hepatocyte apoptosis by reducing the activation of caspases 3 and 8 and returning hepatic stellate cells to a quiescent state.^72^ Pirfenidone also inhibits several other anti‐inflammatory and anti‐fibrogenic pathways in animal models, such as the profibrogenic Th2 response.^13^ Furthermore, extended‐release pirfenidone shows promise in the context of NASH. It has demonstrated a true agonist/ligand relationship with PPAR‐α, providing an anti‐steatogenic effect and offering protection against inflammation and liver fibrosis.^73^, ^74^

In various liver diseases, inflammation is linked to stellate cell activation and subsequent liver fibrosis. Salazar‐Montes et al.^75^ investigated the antioxidant and antifibrotic effects of pirfenidone in a cirrhotic animal model. Their study showed a reduction in fibrotic gene expression, such as TGF‐β and collagen 1‐α, and an increase in regenerative genes like hepatocyte growth factor and c‐met. They also observed a decrease in oxidative gene expression, including superoxide dismutase, catalase, iNOS, and nuclear factor kappa B (NFκB), concluding that pirfenidone has significant antifibrotic and antioxidant effects. Nakanishi H et al.^76^ demonstrated that pirfenidone inhibits the induction of iNOS mRNA and protein, reducing nitric oxide production via NFκB inhibition, through the hepatocyte IL‐1 receptor. Garcia L et al.^77^ showed that pirfenidone reduced the gene expression of collagens I, III, and IV, TGF‐β1, Smad‐7, TIMP‐1, and plasminogen activator inhibitor‐1 in animal models. Histological analysis of pirfenidone‐treated rats indicated a 50% reduction in liver fibrosis, decreased hydroxyproline levels, and reduced type 1 collagen mRNA expression, highlighting pirfenidone's inhibitory effect on collagen production in stellate cells.

The impact of pirfenidone on hepatocellular carcinoma (HCC) has been investigated, suggesting potential tumor‐suppressive effects. Pirfenidone may reduce fibrosis, inflammation, and promote apoptosis in HepG2 cells.^78^, ^79^ Experimental models have shown that pirfenidone inhibits HCC cell proliferation and suppresses β‐catenin expression in HepG2 cells. Zou et al. demonstrated that pirfenidone inhibits the Wnt/β‐catenin signaling pathway, preventing HCC cell proliferation.^79^ These findings suggest that pirfenidone may influence fibrosis, inflammation, apoptosis, and cell proliferation in HCC. Silva‐Gomez et al.^78^ provided further evidence of pirfenidone's effectiveness in preventing histological damage associated with TGF‐β1 and α‐SMA expression in animal models. The observed reduction in IKK and IkB‐phosphorylation/NFkB p65 expression and translocation supports its potential tumor‐suppressive effects, making pirfenidone a candidate therapeutic agent for HCC. However, no clinical trial has been published on the use of pirfenidone in patients with HCC.

Few studies have explored the effect of pirfenidone on viral hepatitis. Flores‐Contreras et al. examined its impact on 34 patients with chronic hepatitis C, administering 1200 mg of pirfenidone daily for 24 months. They found improvements in histological steatosis, necroinflammation, and fibrosis, along with reduced serum TGF‐β1 and IL6 levels, and increased antifibrogenic CB2 receptor gene expression.^80^ Another pilot study investigated pirfenidone's effects on 15 patients with chronic hepatitis C. It was well‐tolerated and improved histological activity scores in 53.3% of patients, along with reductions in steatosis (60%) and fibrosis (30%), and increased liver cell regeneration in 70% of patients. Despite severe chronic liver disease, there were no adverse effects on transaminases. However, further verification is needed through double‐blind, placebo‐controlled clinical trials.^81^

In the metabolic associated steatotic liver disease (MASLD)/NASH experimental model, the effect of pirfenidone has been studied, revealing modifications in the metabolic pathways of genes related to lipid metabolism, insulin resistance, and inflammatory response.^73^, ^82^ Escutia‐Gutierrez et al. demonstrated a reduction in the expression of hepatic miRNAs and target genes involved in inflammation (IL1b, TNF‐α, IL6, TGFβ1), lipid SrebF1, and Col1a1 synthesis.^82^ Moreover, Chen G et al. investigated the effect of pirfenidone in NASH‐induced rats, observing attenuated lipid accumulation and peroxidation by reducing lipogenesis and fatty acid synthesis in rats exposed to pirfenidone.^83^ They also demonstrated a reduction in the number of hepatic CD11c + CD206‐(M1) macrophages and T cells, contributing to the amelioration of steatohepatitis and insulin resistance.^83^ A pilot study explored the effect of pirfenidone in primary sclerosing cholangitis (PSC), showing no benefit in PSC patients and frequently associated adverse effects.^84^ Nevertheless, the use of pirfenidone in liver fibrosis remains limited to small sample size and underpowered trials, necessitating double‐blind, randomized, placebo‐controlled clinical trials to verify its effect.^85^

### 5.5 Ophthalmologic disorders

Pirfenidone has been tested for proliferative vitreoretinopathy, which is a major sequel of penetrating or open globe injury. In an animal model, intravitreal pirfenidone prevented the expression of alpha‐smooth muscle, TGF‐β, and collagen‐1, as well as the inhibition of proinflammatory cytokine secretion.^86^

In Graves’ ophthalmopathy, Wu et al.^87^ found that pirfenidone inhibits TGF‐β1 phosphorylation of p38 and JNK in fibroblasts, suggesting it modulates TGF‐β1 pathways involved in fibroblast differentiation and extracellular matrix homeostasis. Similarly, Li et al.^88^ demonstrated pirfenidone's antifibrotic effect on orbital fibroblasts by inhibiting cell proliferation, TGF‐β1 expression, and collagen secretion.

In an animal model of glaucoma, Kasar et al.^89^ showed that pirfenidone delays wound healing by inhibiting TGF‐β1 and fibroblast growth factor β (FGF‐β) secretion. Dixon et al.^90^ demonstrated the potential of pirfenidone/vitamin E‐loaded contact lenses to reduce corneal haze after alkali burn in a rabbit model, offering a promising therapy for corneal inflammation and fibrosis.

### 5.6 Skin fibrosis

A randomized clinical trial by Mecott‐Rivera et al. evaluated the effect of topical pirfenidone on healing times in patients with skin grafts after skin burn injuries. They concluded that topical pirfenidone was effective in reducing healing times after split‐thickness skin grafts.^91^ Also, Mecott et al.^92^ propose oral pirfenidone treatment in patients with extensive second‐degree burns, showing a decrease in wound healing time by enhancing wound re‐epithelialization observed with pirfenidone. This effect was confirmed by Wells et al.^93^ in an in vitro model, showing a promising antifibrotic effect in treating scarring and wound healing in burn injuries. The effect of pirfenidone on keloid lesions has been explored. Pirfenidone causes suppression of keloid‐derived fibroblast contraction through inhibition of the TGF‐β1 pathway, demonstrating the potential therapeutic effect of pirfenidone for the treatment of keloid lesions.^94^ Likewise, Armendariz‐Borunda et al. evaluated pirfenidone in hyperproliferative burn scars against conventional pressure therapy, demonstrating that the pirfenidone group had a higher improvement in all scar features compared with the standard treatment group.^95^

In a different context, Rodriguez‐Castellanos et al.^96^ conducted a study with topical pirfenidone in localized scleroderma, showing histopathological improvement in terms of epidermal atrophy, inflammation, dermal or adipose tissue fibrosis, and annex atrophy. The application of pirfenidone gel was well‐tolerated, and no side effects were detected. However, potential photoallergic contact dermatitis and photosensitivity have been described in some patients using topical and oral pirfenidone.^26^

### 5.7 Pancreatic fibrosis and other gastrointestinal diseases

The anti‐inflammatory and antiapoptotic effects of pirfenidone in acute pancreatitis have been demonstrated in murine models.^97^ El‐Kashef D et al. reported reduced lipid peroxidation and increased glutathione and superoxide dismutase levels in pancreatic tissue. These changes were associated with suppressed proinflammatory cytokine secretion and NFkB activity, reduced proapoptotic protein Bax, and increased antiapoptotic protein Bcl2.^97^ Another study found that pirfenidone increased IL‐10 levels, which decreased proinflammatory markers and reprogrammed macrophages to an anti‐inflammatory M2 phenotype, ameliorating acute pancreatitis.^98^ In animal models of chronic pancreatitis, Palathingal et al. demonstrated that pirfenidone reduced collagen secretion, proinflammatory cytokine levels, and fibrosis markers in pancreatic stellate cells.^98^

Pirfenidone's effect on human pancreatic cancer cells was investigated in vitro by Usugi et al., who reported suppression of cell proliferation and induction of G0/G1 cell cycle arrest. This was accompanied by increased p21 expression, suggesting potential antitumor effects.^99^ Kozono et al.^100^ evaluated pirfenidone's impact on pancreatic desmoplasia in vitro and in animal models, concluding that pirfenidone combined with gemcitabine more effectively suppressed tumor growth by inhibiting desmoplasia through upregulation of pancreatic stellate cells.

In colon diseases, pirfenidone's effect on ulcerative colitis was studied in a rat model by Antar et al. They found that pirfenidone inhibited proinflammatory biomarker release, restored oxidant/antioxidant balance, preserved colonic architecture, and protected against ulcerative colitis by modulating TGFβ1/JNK‐1 and caspase 3 pathways.^101^ Sun et al.^102^ demonstrated in an animal model that pirfenidone reduced radiation‐induced intestinal fibrosis, collagen deposition, and SMA through inhibition of TGFβ1/Smad/CTGF signaling pathways. Additionally, pirfenidone was shown to inhibit proliferation and matrix metalloproteinase‐3 production in human intestinal fibroblasts from Crohn's disease patients.^103^

## 6 CONCLUSIONS AND PERSPECTIVES

**Figure 2:**
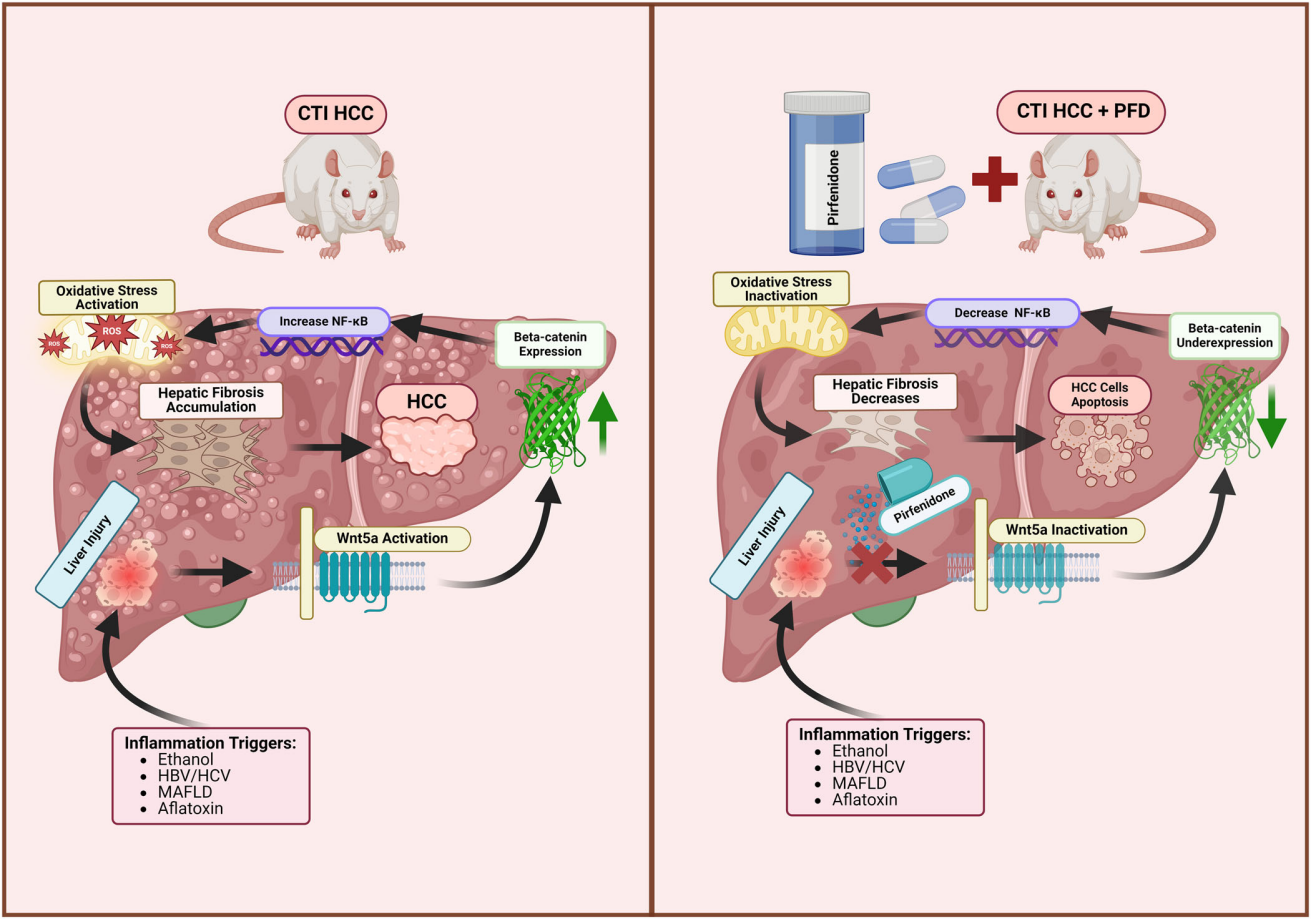
Pirfenidone effect in hepatocarcinogenesis. Hepatocellular carcinoma (HCC) pathophysiology involves the expression of beta‐catenin, which activates NF‐kB, leading to increased oxidative stress. This cascade contributes to hepatic fibrosis and formation of cancerous cells. Pirfenidone (PFD) may act as a tumor suppressor by reducing fibrosis, inflammation, and apoptosis. In models, PFD has been shown to inactivate the Wnt5a signaling pathway. This inactivation suppresses beta‐catenin expression and decreases NF‐kB levels, preventing oxidative stress and the release of proinflammatory cytokines. Consequently, hepatic fibrosis is reduced, and apoptosis of HCC is increased.

**Figure 3:**
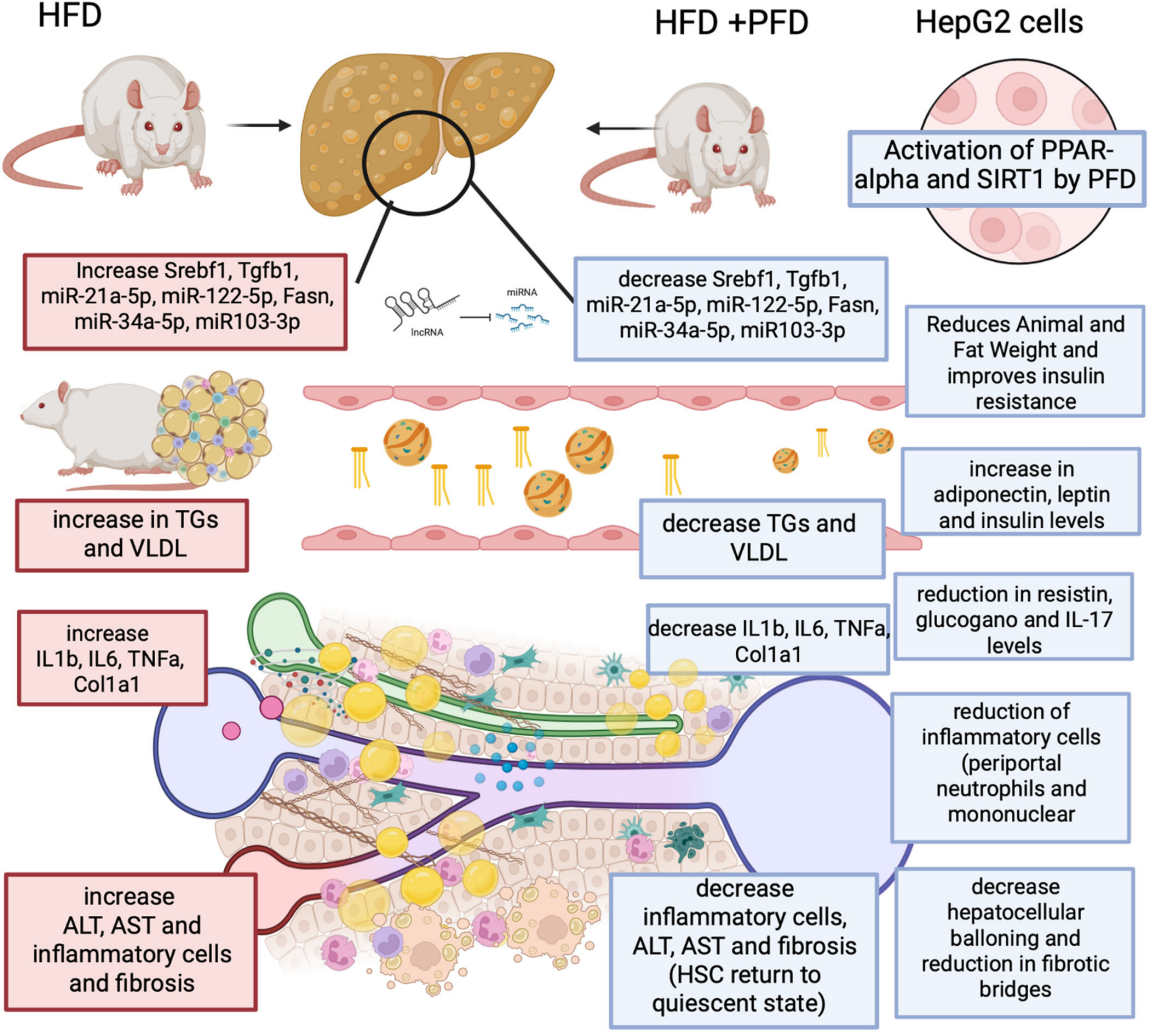
Effect of pirfenidone in metabolic dysfunction‐associated steatotic liver disease in high‐fat diet (HFD) fed mice and HepG2 cells. In HFD‐fed mice, pirfenidone treatment reduces fibrosis and inflammation by decreasing the expression of fibrotic and inflammatory genes and microRNAs, lowering triglycerides (TGs), very low‐density lipoprotein (VLDL), and inflammatory cytokines like IL1b, IL6, and TNFa. It also reduces ALT and AST levels, fibrosis, and inflammatory cells, and promotes the quiescence of hepatic stellate cells (HSC). In HepG2 cells, pirfenidone activates Peroxisome Proliferator‐Activated Receptor (PPAR)‐alpha and Sirtuin 1 (SIRT1), improving insulin resistance, and reducing resistin, glucagon, and IL‐17 levels while increasing adiponectin, leptin, and insulin levels. It also reduces periportal neutrophils and mononuclear cells, demonstrating its broad anti‐inflammatory and antifibrotic effects.

Pirfenidone has demonstrated several anti‐inflammatory and antifibrotic effects with good tolerance, making it a potentially valuable option for treating various fibrotic diseases (Table S1). Several clinical trials are ongoing to demonstrate further evidence (Table S2). Although the use of pirfenidone remains debatable, additional clinical trials are necessary to confirm its efficacy across diverse fibrotic pathologies in different organs (Figures 2 and 3).

## AUTHOR CONTRIBUTIONS

**Aldo Torre**: Conceptualization; Data curation; Investigation; Project administration; Supervision; Validation; Visualization; Writing—original draft. **Froylan David Martínez‐Sánchez**: Data curation; Investigation; Methodology; Project administration; Supervision; Validation; Visualization; Writing—original draft; Writing—review & editing. **Sofía Mercedes Narvaez‐Chávez**: Conceptualization; Data curation; Investigation; Methodology; Supervision; Validation; Writing—original draft; Writing—review & editing. **Mariana Ariel Herrera‐Islas**: Conceptualization; Investigation; Methodology; Validation; Visualization; Writing—original draft; Writing—review & editing. **Carlos Alberto Aguilar‐Salinas**: Investigation; Project administration; Supervision; Validation; Visualization; Writing—original draft; Writing—review & editing. **Jacqueline Córdova‐Gallardo**: Conceptualization; Data curation; Formal analysis; Funding acquisition; Investigation; Methodology; Project administration; Supervision; Validation; Visualization; Writing—original draft; Writing—review & editing. No sources of funding were used to assist in the preparation of this review. The article processing charges for this work were funded by the Universidad Nacional Autónoma de México.

## CONFLICT OF INTEREST STATEMENT

The authors declare no conflict of interest.

## Supporting information

Supplementary information.

Supplementary information.

## Data Availability

Availability of data and material Data sharing is not applicable to this article as no datasets were generated or analyzed during the current review.
