# Supplementary material for: Pirfenidone use in fibrotic diseases: What do we know so far?
Source: Immun Inflamm Dis. 2024 Jul 5;12(7):e1335. doi: 10.1002/iid3.1335 (PMC11225083; doi:10.1002/iid3.1335)
Supplement: Supplementary file 1 — Supplementary information. [file IID3-12-e1335-s002.docx]

**Supplementary table 1. Summary of Clinical trials that used pirfenidone as treatment for fibrotic diseases.**

| **Study** | **Type of study** | **Organ** | **Disease** | **Number of patients** | **Dose and time** | **Effects** | **Adverse effects** |
| --- | --- | --- | --- | --- | --- | --- | --- |
| Benefits of prolonged-release pirfenidone plus standard of care treatment in patients with advanced liver fibrosis: PROMETEO study  [28] | Open labelled | liver | Advanced liver fibrosis | 196 patients (122 pirfenidone +SOC; 74 SOC) 64± | 600mg bid | Improve in Child-Pugh score, reduction of TGFβ1 levels, fibrosis regression. | Transient burning, nausea, photosensitivity |
| Treatment with pirfenidone for two years decreases fibrosis, cytokine levels and enhances CB2 gene expression in patients with chronic hepatitis C.  [80] | Open labelled, non-controlled non-randomized clinical trial | Liver | Hepatitis C | 34 patients  M13:F21  Age 56±10 years | 1200mg/d (400mg tid) for 24 months | Decreases necroinflammatio grade, fibrosis stage and steatosis, reduction of TGF-β1 and IL-6 and increase in gene expression of CB2 receptor | Gastritis (81%), nauseas (48%), rash (29%), photosensitivity (14%), vomiting (5%), dissiness (10%), weakness (10%), insomnia (5%), somnolence (5%) |
| A pilot study in patients with established advanced liver fibrosis using pirfenidone [81] | Pilot clinical trial | Liver | Hepatitis C | 15 patients (57 years (48-70) | 1200mg/d | necroinflammatory  reduction (53.3%), Steatosis reduction (60%), Fibrosis reduction (30%), Liver cell regeneration (70%) | photosensitivity, rash and itching, and gastrointestinal symptoms (nausea, abdominal discomfort, and diarrhea)  15% |
| Pirfenidone in the treatment of primary sclerosing cholangitis  [84] | Pilot clinical trial | Liver | PSC | 24 patients | 2400mg/d | No improvement in Mayo risk score neithet in histological (inflammation, fibrosis, histologic stage disease) nor in cholangiographic findings | 83% present adverse effects |
| Pirfenidone increases the epithelialization rate of skin graft donor sites  [91] | Randomized controlled trial | skin | Skin graft in burned patients | 24 patients 21 years (5-73) | Topical pirfenidone | Pirfenidone is efficient in reducing the healing times | None |
| Pirfenidone exerts beneficial effects in patients with IPF undergoing single lung transplantation.  [42] | Open labelled, non-controlled non-randomized clinical trial | Lung | Lung transplantation for Idiopathic pulmonary fibrosis | 17 patients (62.7±2.7 years) | Oral (dose not specified taken the day before and the day of transplantation | Reduction in primary graft dysfunction, length of mechanical ventilation and reduction of incidence of acute cellular rejection in the first 30 days | Not described |
| Pirfenidone in patients with idiopathic pulmonary fibrosis (CAPACITY): two randomised trials  [30] | Randomized controlled trial | Lung | Idiopathic pulmonary fibrosis | 174 patients  87 patients  Age 40-80 years) | 2403mg/day  1197mg/day  For 72 weeks | Not difference in FVC  however, a consistent pirfenidone effect was apparent until week 48 | Nausea, dyspepsia, vomiting, anorexia, photosensitivity, rash, dizziness. |
| Pirfenidone in patients with idiopathic pulmonary fibrosis and more advanced lung function impairment  [31] | Randomized controlled trial | Lung | Idiopathic pulmonary fibrosis | 90 patients | 2403mg/day | Lower risk of all-cause mortality, ≥10% absolute %FVC decline or respiratory-related hospitalisation. | 14.4%, Gastrointestinal or skin related. |
| Pirfenidone in patients with rapidly progressive interstitial lung disease associated with clinically amyopathic dermatomyositis  [41] | Open label, prospective study, with matched retrospective controls | Lung | Interstitial lung disease (ILD) associated with amyopathic dermatomyositis | 30 patients | 1800mg/day | Lower mortality, impact in the survival of subacute ILD patients | Elevations of hepatic enzimes (30%), gastrointestinal reaction (13.3%) mild to moderate.  Severe reactions (10%) leading to interruption (rash, diarrhea and important elevation of LFT) |
| Pirfenidone in patients with progressive fibrotic interstitial lung diseases other than idiopathic pulmonary fibrosis (RELIEF): a double-blind, randomised, placebo-controlled, phase 2b trial  [40] | multicentre, double-blind, randomised, placebo-controlled, parallel phase 2b trial | Lung | fibrotic interstitial lung diseases other than IPF | 127 patients (64 pirfenidone and 63 placebo)  18-80 years | 267 mg three times per day in week 1, 534 mg three times per day in week 2, and 801 mg three times per day thereafter | Adding pirfenidone to standard treatment might attenuate disease progression with a decline in FVC | nausea (2 pirfenidone, 2 placebo), dyspnea (1 pirfenidone, 1 placebo), and diarrhoea (1 pirfenidone) |
| Pirfenidone slows renal function decline in patients with focal segmental glomerulosclerosis  [68] | open-label trial | Kidney | focal segmental glomerulosclerosis | 21 patients | 800 mg three times daily | GFR improved in 25%, not effect in proteinuria. | dyspepsia, sedation, and photosensitive dermatitis |
| Pirfenidone for diabetic nephropathy  [69] | Randomized controlled trial | Kidney | diabetic nephropathy | 77 patients (26 placebo/ 51 pirfenidone) | 25 patients in pirfenidone group (2400 mg/d) ;  26 patients in placebo group (1200 mg/d) | eGFR change not significantly different from placebo, Hemodialysis 4 in placebo, 1 pirfenidone 2400-mg/d group, and none in the pirfenidone 1200-mg/d group. | gastrointestinal, fatigue, and photosensitivity rash |
| Pirfenidone in heart failure with preserved ejection fraction: a randomized phase 2 trial  [55] | Randomized, doble-blind phase II | Heart | heart failure | 12 patients pirfenidone, 14 placebo | 2403mg per day | administration of pirfenidone for 52 weeks reduced myocardial fibrosis | nausea, insomnia and rash |
