# Supplementary material for: Pirfenidone use in fibrotic diseases: What do we know so far?
Source: Immun Inflamm Dis. 2024 Jul 5;12(7):e1335. doi: 10.1002/iid3.1335 (PMC11225083; doi:10.1002/iid3.1335)
Supplement: Supplementary file 2 — Supplementary information. [file IID3-12-e1335-s001.docx]

**Supplementary table 2. Clinical trials currently in course (clinicaltrials.gov).**

| **Study** | **Title** | **Type of study** | **Phase** | **Target organ** | **Patients and Disease** | **Dose** | **Completion date** |
| --- | --- | --- | --- | --- | --- | --- | --- |
| NCT05505409 [104] | Efficacy, Safety, Immune Function of Pirfenidone in the Treatment of Connetive Tissue Disease -Related Interstitial Lung Disease (CTD-LID). | Randomized- parallel assignment, Open Label | Phase 4 | Lung and connective tissue | 120 patients  (18 years and older) | NS | December 1, 2025 |
| NCT05280873 [105] | Pirfenidone Combined With Methylprednisolone Versus Methylprednisolone in the Treatment of CIP | Randomized, parallel assignment, open label | Phase 1 | Lung | 48 patients (18 to 75 years) | Methylprednisolone 2 mg / kg / d+ pirfenidone (starting from 200mg tid, increasing to 600mg tid within one week and maintaining) | October 20, 2024 |
| NCT05075161 [106] | Pirfenidone to Prevent Fibrosis in Ards. (PIONEER) | Randomized, parallel assignment. Quadruple masking | Phase 3 | Lung | 130 patients  (18 years and older) | 801mg/day; from days 8-14:1602mg/day, from day 15 to ICU discharge 2403 mg/day. All drugs will be delivered by a nasogastric tube divided in 3 daily doses. | October 2025 |
| NCT03939520 [107] | Management of Progressive Disease in Idiopathic Pulmonary Fibrosis (PROGRESSION) | Randomized, parallel assignment, open label | Phase 4 | Lung | 378 patients (50 years and older) | pirfenidone 2403 mg per day (at least 1602 mg) in combination with nintedanib 300 mg per day (at least 200 mg) during 24 weeks. | December 2022 |
| NCT04928586 [108] | Immunosuppressant Combined With Pirfenidone in CTD-ILD | Non-Randomized, parallel assignment, triple blinding | Phase 4 | Lung | 200 patients (18-80 years) | pirfenidone up to the maximum tolerable dose | June 1, 2025 |
| NCT04607928 [109] | Pirfenidone Compared to Placebo in Post-COVID19 Pulmonary Fibrosis COVID-19 (FIBRO-COVID) | Randomized, parallel assignment, single masking | Phase 2 | Lung | 148 patients  (18 years and older) | NS | June 30, 2022 |
| NCT04856111 [110] | Pirfenidone vs. Nintedanib for Fibrotic Lung Disease After Coronavirus Disease-19 Pneumonia (PINCER) | Randomized, parallel assignment, single masking | Phase 4 | Lung | 48 patients (18 years and older) | Pirfenidone will be started at a dose of 600 mg/day. The dose will be escalated by 600 mg/day every 3-7 days up to a targeted dose of 2400 mg/day. The subjects will be administered the maximum tolerated dose for a total period of 24 weeks from randomization. | June 30, 2022 |
| NCT05118256 [111] | Pirfenidone for the Reduction of Metabolic, Inflammatory and Fibrogenic Activity in Complicated Silicosis | Randomized, parallel assignment, open label | Phase 2 | Lung | 18 patients (18 years and older) | NS | November 15, 2023 |
| NCT05542615 [112] | Prolonged Release Pirfenidone for Advanced Residual Liver Fibrosis (MINERVA). | Single Group assignment open label | Phase 2 | Liver | 60 patients (18 years and older) | 1200 mg / day of Pirfenidone | December 1, 2023 |
| NCT04126538 [113] | Pirfenidone Capsule in Patients With Chronic Kidney Disease G2 and G3a Study on Safety and Pharmacokinetics | Non-Randomized, parallel assignment, open label | Phase 1 | Kidney | 24 patients  (18 years and older) | 400mg per day | June 30, 2022 |
| NCT04258397 [114] | Trial of Pirfenidone to Prevent Progression in Chronic Kidney Disease (TOP-CKD) | Randomized, parallel assignment, triple masking | Phase 2 | kidney | 200 patients (21 years and older) | 5 capsules (1335 mg pirfenidone): 2 pills in the morning, 1 mid-day, and 2 in the evening, with meals. | December 2024 |
